# A state-of-the-science review and guide for measuring environmental exposure biomarkers in dried blood spots

**DOI:** 10.1038/s41370-022-00460-7

**Published:** 2022-08-13

**Authors:** Tyler A. Jacobson, Jasdeep S. Kler, Yeunook Bae, Jiexi Chen, Daniel T. Ladror, Ramsunder Iyer, Denise A. Nunes, Nathan D. Montgomery, Joachim D. Pleil, William E. Funk

**Affiliations:** 1 Department of Preventive Medicine, Northwestern University Feinberg School of Medicine, Chicago, IL USA; 2 University of Michigan Medical School, Ann Arbor, MI USA; 3 Galter Health Sciences Library, Northwestern University Feinberg School of Medicine, Chicago, IL USA; 4 Department of Environmental Sciences and Engineering, Gillings School of Public Health, University of North Carolina, Chapel Hill, NC USA

**Keywords:** Dried blood spots, Biomarkers, Environmental Tobacco Smoke, Persistent organic pollutants, Trace elements, Biomonitoring

## Abstract

**Background:**

Dried blood spot (DBS) sampling is a simple, cost-effective, and minimally invasive alternative to venipuncture for measuring exposure biomarkers in public health and epidemiological research. DBS sampling provides advantages in field-based studies conducted in low-resource settings and in studies involving infants and children. In addition, DBS samples are routinely collected from newborns after birth (i.e., newborn dried blood spots, NDBS), with many states in the United States permitting access to archived NDBS samples for research purposes.

**Objectives:**

We review the state of the science for analyzing exposure biomarkers in DBS samples, both archived and newly collected, and provide guidance on sample collection, storage, and blood volume requirements associated with individual DBS assays. We discuss recent progress regarding analytical methods, analytical sensitivity, and specificity, sample volume requirements, contamination considerations, estimating extracted blood volumes, assessing stability and analyte recovery, and hematocrit effects.

**Methods:**

A systematic search of PubMed (MEDLINE), Embase (Elsevier), and CINAHL (EBSCO) was conducted in March 2022. DBS method development and application studies were divided into three main chemical classes: environmental tobacco smoke, trace elements (including lead, mercury, cadmium, and arsenic), and industrial chemicals (including endocrine-disrupting chemicals and persistent organic pollutants). DBS method development and validation studies were scored on key quality-control and performance parameters by two members of the review team.

**Results:**

Our search identified 47 published reports related to measuring environmental exposure biomarkers in human DBS samples. A total of 28 reports (37 total studies) were on methods development and validation and 19 reports were primarily the application of previously developed DBS assays. High-performing DBS methods have been developed, validated, and applied for detecting environmental exposures to tobacco smoke, trace elements, and several important endocrine-disrupting chemicals and persistent organic pollutants. Additional work is needed for measuring cadmium, arsenic, inorganic mercury, and bisphenol A in DBS and NDBS samples.

**Significance:**

We present an inventory and critical review of available assays for measuring environmental exposure biomarkers in DBS and NDBS samples to help facilitate this sampling medium as an emerging tool for public health (e.g., screening programs, temporal biomonitoring) and environmental epidemiology (e.g., field-based studies).

## Introduction

Human biomonitoring has found a prominent role in investigating relationships between environmental exposures and adverse health outcomes. Major government tracking studies, such as the National Health and Nutrition Examination Study (NHANES), have measured key components of the human exposome in blood and urine using biomarker measurements to retrospectively assess exposures, and prospectively interpret disease states on a population level [1, 2]. Myriads of smaller studies have focused on specific links between environmental exposures and disease using combinations of blood, breath, lavage fluids, adipose tissues, and urine as the biological media for informing the exposure to risk paradigm [3–6]. Unlike environmental measurements (e.g., measuring pollutants in air and drinking water), biomarker measurements can be relatively invasive. While medical patients may be willing to provide repeated blood draws and collection of their urine, the general public is not so acquiescent in allowing biological monitoring for indirect purposes of public health assessment. As such, the value of environmental biomonitoring is best supported with the least invasive, simplest sampling methods in the field, with perhaps more complex analyses reserved for the laboratory [7].

Blood analysis has often been considered the “gold standard” for human exposure and disease diagnostics [8]. However, the collection of venous blood is relatively invasive and requires trained medical personnel, costly refrigeration and shipping, and special laboratory processing and handling [9]. Dried blood spot (DBS) samples are 4–5 drops of whole blood from a minimally invasive finger- or heel-prick, absorbed onto specially designed filter paper (e.g., Whatman 903). DBS samples can be shipped at ambient temperatures in flat envelopes [9], since the United States Postal Service considers DBS samples a Nonregulated Infectious Material. DBS samples are also routinely collected from newborns after birth (i.e., newborn dried blood spots, NDBS) to screen for inborn errors of metabolism and other treatable disorders, and many states in the United States permit access to residual NDBS samples for research purposes. As a result, DBS sampling represents a large and invaluable resource for assessing exposures to environmental toxicants. In addition, DBS sampling allows for self-collection [10, 11], which is an important advantage of this approach during the COVID-19 pandemic. Because of these advantages, DBS sampling is particularly well suited for population-based studies involving younger children and infants, such as the Environmental Influences on Child Health Outcomes (ECHO) program [12, 13]. While these advantages have motivated the use of DBS sampling in several recent large-scale health surveys in the US and globally [14–16], the use of DBS sampling for estimating exposures to chemical toxicants in epidemiological research has recently accelerated within the scientific community, with the publication of many new validated environmental biomarker assays [17, 18].

The utility of DBS for newborn screening was first demonstrated by Robert Guthrie for the testing of phenylketonuria in infants in the early 1960s [19]. Since this time, the use NDBS for screening infants for metabolic disorders has greatly expanded, and routine screening is now standard practice for all US hospitals. This process was accelerated by the introduction of tandem mass spectrometry (MS) in the 1990s, which fostered a new era where large panels of biomarkers could be simultaneously measured in a single analysis [20]. In the US, 35 primary health conditions and 26 recommended secondary targets are included in the Nationally Recommended Uniform Screening Panel by the American College of Medical Genetics [21]. Storage policies and conditions for retaining residual NDBS samples, however, differ widely between states. These differences are often centered around the ethical issues of using archived NDBS without informed parental consent. As a result, many states have chosen to not retain and store residual NDBS samples in the interest of preserving patients’ privacy, while other states retain NDBS specimens for extended timeframes which can be used for research purposes [22]. In addition, even when residual NDBS are retained by states, the cost of storing samples is a significant barrier and can result in suboptimal storage conditions (e.g., storage at room temperature and/or without the use of desiccant). Consequently, NDBS samples are more susceptible to factors such as background contamination and sample degradation. In contrast, DBS samples collected in the field are more carefully handled under standardized research conditions to minimize factors that might influence sample quality.

While DBS provide many advantages over venipuncture, measuring biomarkers in DBS samples poses several challenges, including small and variable blood volumes, requirements for continued lab- and field-based quality assurance measures, validation with gold standard, and higher sample complexity compared to plasma/serum samples. In addition, the stability of biomarkers in DBS samples can be an issue and volatile compounds can be lost during the drying process. Many immunoassay-based methods have been developed; however, these assays tend to have high reagent costs and require long development times. Immunoassays may provide the advantages of high sample throughput and analytical sensitivity but can lack biomarker specificity giving rise to measurement error [23]. MS-based assays provide some advantages because they account for some of these challenges. For example, solid phase extraction and chromatographic separation can be coupled with MS to reduce sample complexity [24]. While MS does not necessarily resolve the issue of assay cost, MS-based assays can be easily multiplexed and provide high biomarker specificity.

When discussing recent progress in the DBS field, limitations and challenges associated with quantifying exposure biomarkers in DBS samples must be considered on a biomarker-by-biomarker basis. This is because sensitivity, specificity, stability, and contamination issues can differ greatly between individual biomarkers [25]. Here, we present a state-of-the-science review for measuring biomarkers in DBS to estimate exposures to environmental toxicants. This review is meant to act as a guide for researchers interested in using DBS in environmental health studies, with a focus on protocols that have been extensively developed and well validated. Details on required sample volumes, biomarker stability, and other important details related to sample collection, shipment, and storage are discussed. By identifying key DBS methods categorized by chemical classes of environmental toxicants, we present an inventory of available assays that will guide the use of DBS sampling in population- and community-based research.

## Methods

**Fig. 1:**
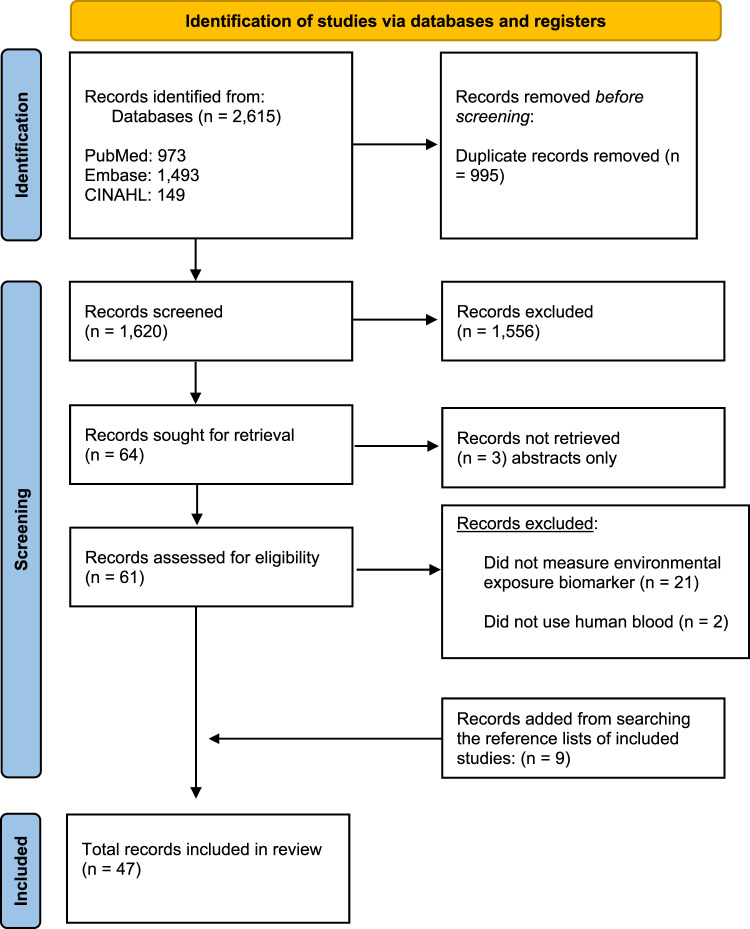
PRISMA flow diagram for identification of studies for final inclusion in the review. PRISMA: Preferred Reporting Items for Systematic Reviews and Meta-Analyses.

**Fig. 2:**
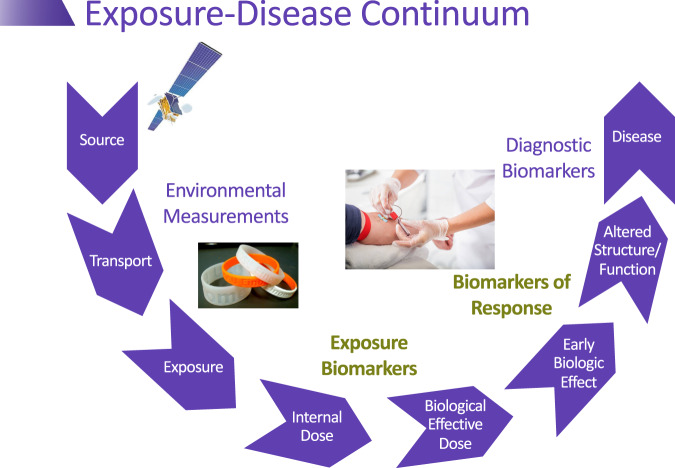
Exposure-disease continuum. This review focuses on environmental exposure biomarkers and excluded studies that used DBS sampling to measure biomarkers of response, for example, non-specific biomarkers of oxidative stress, inflammation, or cholinesterase depression, used commonly in hazard assessments.

In this review, a search of three bibliographic databases was conducted. The search was designed to identify all articles on DBS sampling, biomarkers, and environmental exposures (Fig. 1). A research librarian (DAN) collaboratively developed the search strategies with the review author (TAJ), and on March 7, 2022, searched PubMed (MEDLINE), Embase (Elsevier), and CINAHL (EBSCO). A full list of search strategies and terms is provided in the Supplementary information (SI). Two reviewers (TAJ and JSK) screened the results in duplicate according to pre-determined inclusion criteria using the screening platform, Rayyan. To meet our inclusion criteria (more details: SI), studies had to use DBS sampling to measure biomarkers of internal doses of exposures to exogenous pollutants (i.e., native exogenous compounds and/or their metabolites) in human blood samples. Biomarkers of response were excluded (Fig. 2). DBS method development and validation studies were evaluated based on key quality-control parameters and performance metrics outlined by McDade (2014) [9]. Method development and validation studies were scored by a member of the review team (TAJ, YB, RI, and NDM) and spot checked (TAJ, YB, RI, NDM, and JSK) after being extracted and inputted into Table 1.

**Table 1:** DBS method development studies.

Study (sample type)	Instrument	Precision^a^	Reliability^b^	Accuracy^c^	Sensitivity^d^	Stability^e^	Sample^f^	Notes
*ETS (cotinine)*								
Spector 2007 (NDBS, 1999) [30]	GC-MS				NR	Samples stored for 7 y at 4 °C prior to analyses	¼ of DBS (~12.5 µL^i^)	
Sosnoff and Bernett 2008 (DBS) [33]	LC-MS			X	NR	X (4 y at 4 °C)	6.35-mm discs (~12.5 µL^i^)	
Murphy 2013 (DBS)^g^ [28]	LC-MS	X		*R* = 0.99, *p* < 0.001 (plasma)	LOQ: <0.2 ng/mL^j^	X (10 m at room T)	3.2-mm discs (~3.2 µL^i^) for high exposures; 4.8-mm discs for low exposures (~7.1 µL^i^)	Study 1: 100% DF for plasma cotinine >0.08 ng/mL Study 2: 58.7% DF (comparable to DF % in NHANES) Measured *Trans-3’-HCOT*
Yang 2013 (NDBS, 1999–2003) [31]	LC-MS	X	X	*R* = 0.99, *p* < 0.001 (umbilical cord blood)	LOQ: 3.13 ng/mL	X (7 m at room T)	1 disc × 6.35-mm (~12.5 µL^i^)	23% (100/428) DF amongst all samples 97.8% sensitivity and 98.2% specificity
Searles Nielsen 2014 (NDBS, 2007) [32]	LC-MS	X			Estimated reporting limit: 0.17 ng/mL		2 discs × 6.35-mm (~12.5 µL^i^)	
Tretzel 2016 (DBS) [29]	LC-MS	X	X	X	LOD: 5 ng/mL LOQ: 15 ng/mL	X (30 d at room T)	6-mm diameters (~11.2 µL^i^)	Hematocrit Measured *Trans-3’-HCOT*
Ladror 2018 (DBS)^g^ [34]	LC-MS	X		*R* = 0.94 (plasma)	LOQ: <0.25 ng/mL^j^		3.2-mm discs (~3.2 µL^i^)	100% sensitivity and 94% specificity in predicting smoking status Hematocrit effects negligible
*Lead (Pb)*								
Chaudhuri 2009 (NDBS, NR) [62]	ICP-MS		X	X	0.36 µg/dL^h^	X (8.5 m at room T)	6.35-mm discs (~12.5 µL^i^)	Filter blank contamination: 0.3–0.8 µg/dL
Langer 2011 (NDBS, >7 y) [65]	ICP-MS	X (CV >30%)	X	X	NR^l^		½ DBS (~50 µL^i^)	10% DF
Archer 2012 (NDBS, 2002–2006) [50]	ICP-MS			*R* = 0.48, *p* < 0.0001 (infant BLLs)	NR		3/16-inch discs (~7.0 µL^i^)	
Funk 2013 (NDBS, 2003–2009) [53]	ICP-MS	X	X	X	NR^l^	X (no overall trends across collection years)	½ DBS (~ 30 µL)	100% DF Filter paper contamination: median = 5.7 ppb DBS card acid-cleaned
Funk 2015 (DBS) [52]	ICP-MS	X	X	*R* = 0.99 (venous)	NR^l^		Whole DBS (~50 µL^i^)	DBS card acid-cleaned
Nyanza 2019 (DBS) [48]	ICP-MS	X	X	*R* > 0.9, *p* < 0.0001 (venous)	0.08 µg/L		8-mm diameter (~19.8 µL^i^)	100% DF Field filter blanks: 0.02 ± 0.02 µg/L Lab filter blanks: 0.009 ± 0.017 µg/L
Rodríguez-Saldaña 2021 (DBS)^g^ [46]	TXRF	X	X	*R* = 0.814 in university members and 0.911 in e-waste workers (venous)	LOD: 0.28 µg/dL LOQ: 0.69 µg/dL		DBS whole (~50 µL^i^) or 3-mm discs (~2.8 µL^i^)	Filter paper contamination: ~0.03 ± 0.016 µg/dL
Specht 2021 (DBS) [63]	EDXRF	X		*R* = 0.98, *p* < 0.001 (same sample of whole blood measured by AAS)	1.7 µg/dL		Non-destructive (i.e., whole blood spot card) (~50.0 µL^i^)	Potential hematocrit bias not applicable
*Mercury (Hg)*								
Chaudhuri 2009 (NDBS, NR) [62]	ICP-MS		X	X	LOD: 0.65 µg/L^h^	X (8.5 m at room T)	6.35-mm discs (~12.5 µL^i^)	Undetectable filter paper contamination
Funk 2013 (NDBS, 2003–2009) [53]	ICP-MS	X	X	X	NR^l^	X (no overall trends across collection years)	½ DBS (~30 µL)	33% DF at 1.9 ppb^k^ DBS card acid-cleaned
Funk 2015 (DBS) [52]	ICP-MS	X	X	*R* = 0.98 (venous)	NR^l^		Whole DBS (~50 µL^i^)	DBS card acid-cleaned
Nelson 2016 (NDBS, 4–7 m) [51]	ICP-MS	X		*R* = 0.82 (umbilical cord blood)	T-Hg: 0.7 µg/L		2 discs × 3-mm (~2.8 µL^i^)	Hematocrit effects negligible 38% DF for DBS vs 62% for cord blood
Basu 2017 (1st: DBS, 2nd: NDBS, <14 y)^g^ [64]	GC-CVAFS	X	X	X	Me-Hg: 0.313 µg/L		Batches #1–6: 3-mm discs (~2.8 µL^i^); Batches #7–20: 2-mm × 6-mm rectangular punches (~4.7 µL^i^)	Study 2: 98% DF, comparable to NHANES 2011–2012 Me-Hg assay performance
Nyanza 2019 (DBS) [48]	ICP-MS	X	X	*R* > 0.9 (venous)	T-Hg: 0.012 µg/L		8-mm diameter (~19.8 µL^i^)	100% DF Field filter blanks: 0.006 ± 0.002 µg/L Lab filter blanks: 0.003 ± 0.003 µg/L Used ultrapure HNO_3_ digestion for Hg extraction
Santa-Rios 2020 (DBS)^g^ [47]	GC-CVAFS	X	X	*R* > 0.85 (venous)	Me-Hg: 0.3 µg/L; I-Hg: 1.9 µg/L	X (Me-Hg: 1 y at room T)	Whole DBS (12.7-mm) (~40 µL blood, volume controlled)	Study 2: 94% DF
Schweizer 2021 (DBS) [49]	Direct Hg analysis		X	*R* = 0.95, *p* < 0.001 (venous)	T-Hg: 0.14 µg/L (LOD); 0.28 µg/L (LOQ)	X (4 w at room T and 40 °C)	3 discs × 0.5-inches (~50.0 µL^i^)	
*Cadmium (Cd)*								
Chaudhuri 2009 (NDBS, NR) [62]	ICP-MS		X	X	NR		6.35-mm discs (~12.5 µL^i^)	Low recovery rates (e.g., 53% recovery at lower spiked concentrations)
Langer 2011 (NDBS, >7 y) [65]	ICP-MS	X	X (CV ~50%)	X	NR^l^		½ DBS (~25 µL^i^)	100% or 0% DF depending on statistical method used
Funk 2013 (NDBS, 2003–2009) [53]	ICP-MS	X	X	X	NR^l^	X (no overall trends across collection years)	½ DBS (~30 µL)	67% DF^k^ DBS card acid-cleaned
Funk 2015 (DBS) [52]	ICP-MS	X	X	*R* = 0.94 (venous)	NR^l^	X	Whole DBS (~50.0 µL^i^)	DBS card acid-cleaned
Nyanza 2019 (DBS) [48]	ICP-MS	X	X	*R* > 0.9 (venous)	0.004 µg/L		8-mm diameter (~19.8 µL^i^)	100% DF Field filter blanks: 0.0011 ± 0.001 µg/L Lab filter blanks: 0.001 ± 0.001 µg/L
*Arsenic (As)*								
Funk 2013 (NDBS, 2003–2009) [53]	ICP-MS	X	X	X	NR^l^	X (no overall trends across collection years)	½ DBS (~30 µL blood)	18% DF^k^ DBS card acid-cleaned
Funk 2015 (DBS) [52]	ICP-MS	X	X	*R* = 0.66 (venous)	NR^l^	X	Whole DBS (~50.0 µL^i^)	DBS card acid-cleaned
*EDCs and POPs*								
Burse 1997 (NDBS, 1997) [89]	GC-MS			X	NR		Whole DBS (~50.0 µL^i^)	DDE (p,p’-) only analyte detected
Kato 2009^g^ (NDBS, 2007) [88]	LC-MS	X	X	X	LODs: PFHxS: 0.1 ng/mL; PFOS: 0.4 ng/mL; PFOA: 0.2 ng/mL; PFNA: 0.1 ng/mL	X (up to 61 days at 37 °C)	Whole DBS (~50.0 µL^i^)	100% DF for PFOS and PFOA at concentrations >0.4 ng/mL; 98% DF for PFNA; 70% DF for PFHxS
Ma 2013^g^ (NDBS, 2008–2011) [86]	LC-MS	X (27.0% RSD for PFOS)	X (28.2% RSD for PFOS)	X	LODs: PFOS: 0.03 ng/mL; PFOA: 0.05 ng/mL; BPA: 0.3 ng/mL		16-mm discs (~79.4 µL^i^)	100% DF for PFOS and PFOA; 86% DF for BPA Analyte recoveries low for BPA (~39%) Background contamination: 0.01, 0.1, and 0.6 ng/mL for PFOS, PFOA, and BPA, respectively
Batterman and Chernyak 2014 (DBS) [87]	GC-MS	X		*R* = 0.80 (venous)	LODs: PCB-138, -153, -180: 10, 10, 17 ng/L, respectively; BDE-47, -99: 30 and 30 ng/L, respectively; *p,p’-DDE* (pesticide): 90 ng/L	X (1 m at room T with exception of PBDE; 1 y for refrigeration)	15-mm discs (~69.8 µL^i^)	Background contamination: PCB-180: 35 ng/L; PCB-105: 17 ng/L; PCB-194: 24 ng/L; BDE-47: 35 ng/L; not detectable for other POPs
Poothong 2019 (DBS)^g^ [91]	LC-MS	X	X	*R* values: PFHXs: 0.90 PFOS: 0.97 PFOA: 0.95 PFNA: 0.90 PFDA: 0.72 PFUnDA: 0.94 PFOSA: 0.84 (*p* < 0.0001)	LODs: 0.0075–0.3 ng/mL		10 discs × 3-mm (~2.8 µL^i^)	85% DF for PFHxS, PFOS, PFNA, PFDA, PFUnDA, PFOSA
*Fipronil and metabolites*								
Raju 2016 (DBS) [101]	LC-MS	X	X	X	LODs: Fipronil, Fipronil Sulfone: 0.01 ng/mL; Fipronil desulfinyl: 0.03 ng/mL	X (30 days at room T)	Disc size covering ~10 µL blood	
*Benzene*								
Funk 2008 (NDBS, DBS) [100]	GS-MS	X		*R* = 0.732	NR		NR	Benzene-oxide adducts
*Parabens*								
Mulla 2015 (NDBS) [102]	LC-MS	X	X	X	LOD: 10 ng/mL		Whole DBS (8-mm disc) (~19.8 µL^i^)	55% and 25% DF for MPB and PPB
*Acrylamide*								
Starlin 2020 (DBS) [103]	LC-MS	X	X	X	LOQ: 2.5 µg/mL	X (1 day at −4 °C and room T)	Whole DBS (~50.0 µL^i^)	Internal standard: propranolol

These studies were primarily related to the development and validation of DBS methods for measuring environmental exposure biomarkers.

*AAS* atomic absorption spectroscopy, *BDE* brominated diphenyl ethers, *CV* coefficient of variation, *DF* (%) detection frequency, *EDCs* endocrine-disrupting chemicals, *EDXRF* energy-dispersive X-ray fluorescence, *GC-CVAFS* gas chromatography-cold vapor atomic fluorescence spectrometry, *m* months, *MPB* methyl-parabens, *NR* not reported, *PPB* propyl-parabens, *T* temperature, *TXRF* total reflection X-ray fluorescence, *POPs* persistent organic pollutants, *y* years.

^a^Precision: coefficient of variation, %CV, of a single sample with multiple determinations measured in a single assay.

^b^Reliability: %CV for a single sample with multiple determinations measured on different days.

^c^Accuracy: analyte recovery rates, comparison to matched gold standard (venous blood) or plasma, umbilical cord blood, or infant blood lead levels. Correlation coefficients were reported only when studies regressed matched DBS values to one of the above comparator values.

^d^Sensitivity: limit of detection (LOD), limit of quantification (LOQ), method detection limits (MDLs).

^e^Stability: across different storage conditions, such as temperature, humidity, and time.

^f^Sample requirements: reported in diameter punches and estimated blood volumes (see SI for calculations).

^g^Included an application of the DBS method to measure concentrations of analyte(s) in a population-based study, usually with a relatively small sample size.

^h^Based on Supplementary Table 1 for Rodríguez-Saldaña et al. [46] and Santa-Rios et al. [47].

^i^Blood volume estimates for each disc size were calculated using whole blood applied to a blank filter paper spot. Our careful estimation demonstrates 50 µL of whole blood application corresponds to filling a half-inch (12.7-mm) spot. More details on methods for these calculations are provided in the SI.

^j^Estimated plasma values can be converted to whole blood equivalents by multiplying by 0.58 (i.e., 1 – the average hematocrit for men and women, 42%) (ref. Mayo Clinic. Hematocrit test. 2021. https://www.mayoclinic.org/tests-procedures/hematocrit/about/pac-20384728), assuming no biomarker partitioning across the red blood cell membrane (see discussion in Ladror et al. [34]).

^k^Among a sample from a population with no known exposure(s).

^l^Instrument detection limits, which differ from method or assay detection limits.

X indicated whether the study reported values for this key quality-control assay parameter. For accuracy, the highest-ranking mode of accuracy is listed with correlation coefficients and *p* values if available (for example, matched venous whole blood is considered superior to using reference materials for analyte recovery rates).

## Results

Using the search terms provided in the SI, a total of 2615 reports were found across all databases and relevant reviews. After deduplication, 1620 reports were screened on the basis of titles and abstracts (Fig. 1). The full texts of 61 reports were screened and 23 reports were excluded. Of the reports that did not measure an environmental exposure biomarker, most used DBS sampling to measure non-specific markers of internal biological response (i.e., inflammation, oxidative stress, or cholinesterase depression) to environmental exposures. After full-text review, 38 reports met the inclusion criteria. In addition, 9 reports were identified by searching reference lists of included studies (47 total reports included).

**Fig. 3:**
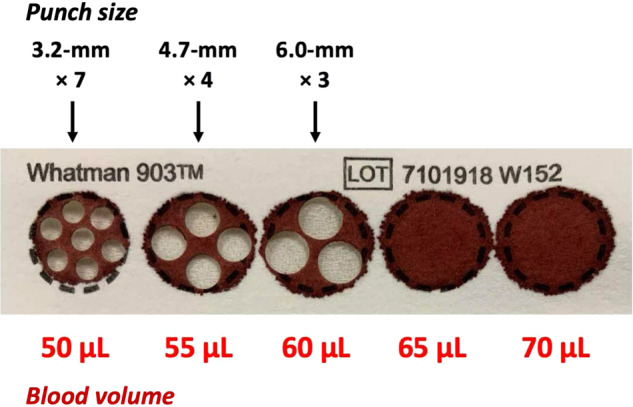
Graphical summary of collection card with dried blood spots. The image shows the size of each spot as a function of the volume of blood applied to the filter paper (50–70 µL). The range between 50 and 70 µL corresponds with the typical volume of a single drop of blood collected by finger- or heel-prick. The punches shown on the first three spots show the number of discs that can be removed based on commonly used disc sizes (i.e., 3.2-mm, ~3.2 µL whole blood; 4.7-mm, ~6.9 µL whole blood; and 6.0-mm, ~11.2 µL whole blood; see Supplementary information for more disc-blood volume estimates).

We highlight key quality-control and performance parameters for each exposure biomarker in Table 1, and we summarize key details from application studies in Table 2. We highlight the estimated blood volumes for different DBS punch sizes in Fig. 3. Of the 47 reports that met our inclusion criteria, 28 reports were categorized as primarily method development and validation (*n* = 7 environmental tobacco smoke (ETS), 12 trace elements, 5 endocrine-disrupting chemicals (EDCs)/persistent organic pollutants (POPs), and 4 other environmental exposure biomarkers) and 19 reports were categorized as being primarily application of previously developed DBS assays (e.g., population-based studies or temporal biomonitoring). However, many method development reports include applications of assays in relatively small sample sizes, while many application-based reports include method and field validation for continued quality assurance.

**Table 2:** Summary of application studies.

Studies	Exposure (biomarker)	Sample size and type	Study design	Main findings	Notes
Joseph 2013 [35]	ETS; cotinine	1541 DBS samples extant child lead screening	Cross-sectional	61% DF; concurrent pediatric screening of lead and ETS using DBS sampling may be feasible	Used DBS assay developed by Murphy et al. (2013) [28]
Spector 2014 [36]	ETS; cotinine	1414 NDBS samples (California, Michigan, New York, Washington)	Cross-sectional	35% DF (83% DF for infants of smoking mothers); evidence of non-disclosure and exposure to SHS during pregnancy	Used DBS assay developed by Murphy et al. (2013) [28]
Sen 2015 [72]	Pb; epigenetic alterations	43 DBS samples from children	Cross-sectional	Elevated Pb exposure associated with alterations in epigenetic profiles	DNA was extracted from DBS samples
Sen 2015 [71]	Pb; epigenetic alterations	35 mother–infant NDBS pairs (Michigan)	Multigenerational cohort study	Elevated Pb levels in NDBS samples from mothers were associated with epigenetic alterations in the child’s NDBS samples	DNA was extracted from DBS samples
Montrose 2020 [73]	Pb; epigenetic alterations	96 NDBS samples (Michigan)	Cross-sectional (within Healthy Families Project – cohort study)	Elevated Pb exposure associated with alterations in epigenetic profiles; DBS sampling suitable for advancing environmental epigenetics	DNA was extracted from DBS samples
Nyanza 2019 [14]	Hg; T-Hg	1056 DBS samples (Tanzanian ASGM communities)	Cross-sectional (Mining and Health longitudinal cohort study)	Although T-Hg levels were higher in pregnant women from ASGM communities compared to non-ASGM, T-Hg levels were elevated in both	Used DBS assay developed by Nyanza et al. (2019) [48]
Nyanza 2020 [15]	Hg; T-Hg	961 DBS samples (Tanzanian ASGM communities)	Mining and Health longitudinal cohort study	Prenatal exposure to Hg was associated with adverse birth outcomes among women in ASGM communities	Used DBS assay developed by Nyanza et al. (2019) [48]
Nyanza 2021 [16]	Pb, T-Hg, Cd	439 DBS samples (Tanzanian ASGM communities)	Mining and Health longitudinal cohort study	High prenatal exposure to Hg was associated with worse neurodevelopment outcomes at 6–12 months of age; Hg and Pb co-exposure may further increase risk	Used DBS assay developed by Nyanza et al. (2019) [48]
Santa-Rios 2020 [69]	I-Hg; Me-Hg	35 DBS samples (Colombian ASGM communities)	Cross-sectional	Me-Hg and I-Hg detected in nearly all samples; field blank filter card contamination was estimated to average ~0.07 ± 0.15 and ~1.16 ± 0.79 µg/L for Me-Hg and I-Hg, respectively. Sample field blanks averaged 0.15 ± 0.19 and 1.77 ± 4.06 µg/L for Me-Hg and I-Hg, respectively. There is a need for Hg speciation	Used DBS assay developed by Santa-Rios et al. (2020) [47]
Santa-Rios 2021 [70]	Me-Hg	20 electronic waste workers (Ghana)	Cross-sectional	Me-Hg detected in nearly all samples; excellent agreement with venous blood values; Me-Hg contamination was low in contaminated field setting	Used DBS assay developed by Santa-Rios et al. (2021) [47]
Spliethoff 2008 [92]	PFOS, PFOSA, PFHxS, PFOA, PFNA	110 pooled NDBS sample composites representing 2640 infants (New York state)	Temporal biomonitoring (1997–2007)	PFOS, PFOSA, PFHxS, PFOA showed exponential declines after the year 2000; DF >90% for all analytes	Included initial method development and validation using spiked DBS samples Pooled composite samples were 24 ×;6-mm diameter punches (~322 µL blood)
Ma 2013 [93]	PBDE congeners	51 pooled NDBS sample composites representing 1224 infants	Temporal biomonitoring (1997–2011)	PBDE exposure declined after 2004; DF 86%, 45%, and 43% for BDEs-47, -99, and -100, respectively. LOQs 0.003, 0.008, 0.008 ng/mL, respectively	Included initial method development and validation using spiked DBS samples Pooled samples were equivalent to 24 ×;6-mm diameter DBS punches (~322 µL blood)
Ma 2014 [90]	PCBs and OCPs	51 pooled NDBS sample composites representing 1224 infants	Temporal biomonitoring (1997–2011)	PCBs and *p,p’-DDE* significantly declined from 1997 to 2001 with *p,p’-DDE* showing continued significant declines through 2011; >50% DF for 12 PCBs and 2 OCPs	Included initial method development and validation using spiked DBS samples Pooled samples were equivalent to 24 × 6-mm diameter DBS punches (~322 µL blood)
Bell 2018 [94]	PFOS, PFOA, BPA	3111 NDBS samples (Upstate KIDS study, 2008–2010)	Longitudinal birth cohort study	PFOS, PFOA not associated with birth size independent of plurality; BPA negatively associated with birth size in twins. 99% DF for PFOS and 90% for BPA	Used DBS assay developed by Ma et al. (2013) [86]
Ghassabian 2018 [96]	PFOS, PFOA, BPA	788 NDBS samples (Upstate KIDS study, 2008–2010)	Longitudinal birth cohort study	Elevated PFOS related to behavioral difficulties; elevated PFOA related to difficulties in prosocial behavior. PFOS and PFOA had DF of 100%	Used DBS assay developed by Ma et al. (2013) [86]
Yeung 2019 [95]	PFOS, PFOA, BPA	3111 NDBS samples (Upstate KIDS study, 2008–2010)	Longitudinal birth cohort study	PFOS and PFOA associated with lower BMI at 3 years of age; postnatal BPA exposure may occur in the hospital	Used DBS assay developed by Ma et al. (2013) [86]
Robinson 2021 [97]	PFOS, PFOA, epigenetic alterations	597 NDBS samples (Upstate KIDS study, 2008–2010)	Cross-sectional	High concentrations of PFOA/PFOS were not clearly associated with significant epigenetic alterations	Used DBS assay developed by Ma et al. (2013) [86] DNA was extracted from DBS samples
Bell 2019 [97]	PCBs, PBDE, *p,p’-DDE*	2065 NDBS samples (Upstate KIDS study, 2008–2010)	Cross-sectional	Elevated POPs were associated with an increased risk for large for gestational age and higher birth weight. >96% DF for DDE and many PCBs using pooled samples	Used DBS methods developed by Ma et al. (2014) [90] and Batterman and Chernyak (2014) [5, 87] DBS samples pooled for analyses
Gross 2020 [99]	OCPs, PBDEs, PFASs	98 NDBS samples (Starting Early Program RCT)	Nested case–control study	>94% DF for most analytes. Two PFASs associated with lower birth weight	Used DBS methods developed by Ma et al. (2013) [86] and Ma et al. (2014) [90]

These studies primarily applied previously developed DBS assays to population-based studies and may contain components of both field and laboratory method development and validation.

*ASGM* artisanal and gold mining, *BPA* bisphenol A, *BDE*brominated diphenyl ethers, *DF* detection frequency, *OCP* organochlorine pesticides, *p,p’-DDE* p,p’-dichlorodiphenyldichloroethylene, *PBDE* polybrominated diphenyl ethers, *PCBs* polychlorinated biphenyl congeners, *PFAS* perfluoroalkyl substances, *PFHxS* perfluorohexane sulfonate, *PFOA* perfluorooctanoic acid, *PFOSA* perfluorooctane sulfonamide, *PFNA* perfluorononanoic acid, *PFOS* perfluorooctane sulfonic acid, *RCT* randomized controlled trial.

### Environmental tobacco smoke

Exposure to ETS poses significant health risks for infants and children, including decreased lung growth, and increased risk of respiratory infections, otitis media, and childhood asthma [26]. Second-hand tobacco smoke (SHS) exposure among non-smokers can also result in adverse health outcomes, including cardiovascular disease and lung cancer, with higher exposure-response relationships at lower levels of exposure [27]. Previous methods for estimating prenatal and postnatal exposures to ETS have relied on administering questionnaires to parents [26], which is subject to both recall and social desirability biases.

Cotinine, a primary metabolite of nicotine, is a sensitive and specific biomarker of exposure to first- and second-hand tobacco smoke and can be quantified in DBS samples. While nicotine has a biological half-life of less than 3 h, cotinine has a biological half-life of 15–20 h [28]. Thus, cotinine persists in the blood stream for longer than nicotine and is the gold standard biomarker for exposure to ETS in blood. Cotinine is further metabolized by P450 2A6 to trans-3’-hydroxycotinine (3’-HCOT) at rates that vary across people. Consequently, some investigators have used the ratio of 3’-HCOT to cotinine to account for variability in nicotine metabolism [28, 29].

#### Overview

A total of nine studies (seven published reports) were conducted on methods development and validation. Of these, six reports were from the US and one was from Germany. Three studies used NDBS samples [30–32] and six studies used DBS samples [28, 29, 33, 34]. Eight studies used human participant samples and one used reference materials with human blood from volunteers [29]. Two studies measured cotinine in matched plasma samples [28, 34] and one study used NDBS and matched umbilical cord blood samples [31]. Four studies reported detection frequencies [28, 31, 34] and two reported sensitivity and specificity in accurately predicting maternal smoking status [31, 34]. These assays were applied in two larger-scale studies involving 1541 DBS samples collected from children during routine lead screening [35] and 1414 archived NDBS samples collected from several states [36].

#### Methods development

Early methods for quantifying cotinine in DBS were not sensitive enough to detect or quantify fetal exposure to SHS [30, 31, 33]. In 2013, a revised method using liquid chromatography-mass spectrometry (LC-MS) was described, with greater analytical sensitivity and precision (limit of quantification (LOQ) of 0.3 ng/g)) and excellent correlation with plasma cotinine levels [28]. This revised method normalized measurements according to excised DBS mass to reduce variability due to hematocrit effects and included quantification of 3’-HCOT to account for variations in nicotine metabolism [28]. This study also analyzed the effects of storage time and conditions on cotinine measurements by comparing subsets of samples stored at either −20 °C or room temperature (20 °C), 11–26 months apart. The study reported no effects of storage time or condition on cotinine measurements [28]. Another assay using LC-MS was validated (LOQ of 3.13 ng/mL) with a strong correlation between cotinine levels in archived NDBS samples and in umbilical cord blood with high sensitivity and specificity in predicting maternal smoking status shortly before birth [31]. This study also reported negligible effects of storage time and conditions on cotinine measurements by analyzing subsamples stored in dark and room temperature for 7 months [31]. Although NDBS values were highly correlated with umbilical cord blood cotinine levels, they were on average 15.5 ng/mL lower [31]. This bias was more pronounced when DBS samples were collected >2 days after birth [31]. An automated extraction procedure to enable high throughput analyses of nicotine, cotinine, and 3’-HCOT has also been described [29]. This study reported negligible hematocrit effects for levels ranging from 30 to 60% [29].

Recently, an ultra-sensitive (LOQ < 0.25 ng/mL), high throughput method for quantifying cotinine in plasma and reconstituted DBS samples of smokers and non-smokers was developed [34]. This method utilized a single 3.2-mm DBS punch (estimated ~5 µL blood) and used DBS-based calibration standards to account for matrix effects [34]. DBS cotinine levels were highly correlated with matched plasma samples and had high sensitivity and specificity in distinguishing smokers from non-smokers [34]. Hematocrit effects were negligible [34]. This assay was applied to 50 archived DBS samples (with unknown smoking status) collected via finger-prick from infants and children ages 0–21 years old [34]. In total, 7 out of the 50 samples had levels of cotinine above the assay’s LOQ [34].

#### Guidance

The assays developed by Ladror et al. [34] and Murphy et al. [28] have the highest sensitivities (LOQ ~0.25 ng/mL) and require the least amount of sample volumes (e.g., 3.2-mm punches for high-exposure groups or 4.8-mm punches for low-exposure groups). These assays have been validated on key quality-control metrics (Table 1) and have been developed for high sample throughput. This level of analytical sensitivity is sufficient to quantify the 90th percentile of serum cotinine among non-smokers in the US (0.305–0.356 ng/mL) [37]. Hematocrit effects have been investigated by three studies and have been found to be negligible [29, 33, 34]. Cotinine concentrations were reported to be stable in DBS and NDBS samples for at least 7–10 months at room temperature [28, 31] and up to 4 years at 4 °C (small sample size) [33]. Additional research is needed into potential matrix effects, including whole blood (DBS) versus plasma/serum [34]. Duplicate testing on positive DBS values <10 ng/mL is recommended to minimize false positive results [31]. The optimal point on the receiver operating curve to differentiate active smoking versus non-smoking status in DBS samples is 6 ng/mL [31]. Based on the high analytical sensitivities, sample throughput, small sample volume requirements, and high-quality-control parameters of developed assays for measuring cotinine in DBS samples, these assays appear ready for use in large-scale population-based studies and public health screening programs.

#### Applications

DBS approaches have been applied to two large pediatric cohort studies to detect cotinine in extant DBS samples collected during routine lead screening [35, 36]. Both of these studies used a previously described and validated assay with a limit of detection (LOD) of 0.3 ng/g (~0.2 ng/mL blood) [28]. Significantly higher cotinine levels were independently associated with African American race, older age, Medicaid coverage, higher state smoking rates, and higher average winter temperatures [35]. Cotinine levels were detected in 61% of DBS samples and were strongly associated with elevated blood lead levels in DBS samples [35].

The assay developed by Murphy et al. was also applied to an observational, cross-sectional study with a large collection of newborn DBS samples from screening programs in California, Michigan, New York, and Washington [36]. Cotinine levels (>0.3 ng/g) were detected in 35% of newborn DBS samples, and higher levels were associated with African American race due to environmental racism, racist advertising policies, and residential segregation [36, 38]. This study also found evidence of non-disclosure among mothers: cotinine levels suggesting active smoking status of the mother (>9.0 ng/g) were found in 12% of NDBS samples, despite 41% of these mothers reporting that they did not smoke during pregnancy [36]. These findings support bias in self-report smoking data, which would underestimate the true impact of ETS exposure on health outcomes.

### Trace elements

Prenatal and childhood exposure to trace elements, including arsenic (As), lead (Pb), mercury (Hg), and cadmium (Cd), are a significant public health concern. Here, we focus on As, Pb, Hg, and Cd because they are listed as the first, second, third, and seventh most hazardous substances on the Agency for Toxic Substances and Disease Registry’s 2019 CERCLA priority list of 275 substances, respectively. Exposure to Pb, Cd, and As has also been implicated in the progression of cardiovascular disease [39] and chronic exposure to low levels of Pb has been linked to cognitive and behavioral disturbances in children [40]. Up until 2012, children were identified as having a blood lead “level of concern” with values >10 µg/dL [41]. The CDC has revised its guidelines to consider any value >3.5 µg/dL a blood lead “reference value” that puts the child in the 97.5th percentile of blood lead levels among US children 1–5 years old [41]. This value is not health-based, and there is no established safe level of lead exposure in children.

Exposures to Hg, As, and Cd are also a major health concern and deserve special attention. Human exposure to methyl-Hg occurs primarily through the dietary consumption of marine fish and other seafood. Methyl-Hg readily crosses the placenta and passes through the fetal blood–brain barrier. Chronic, low-level exposure to methyl-Hg, especially in utero and in the first 2 years of life, may increase the risk for neurologic and psychiatric conditions later in life [42, 43]. Human exposure to As may occur through drinking from contaminated water sources and from dietary consumption. Arsenic has been associated with an increased risk for cancers of the skin, lung, bladder, kidney, and liver—with early-life susceptibility [44]. Similarly, Cd exposure occurs primarily through consumption of contaminated food and water, as well as from the inhalation of cigarette smoke [45]. Observational studies have linked Cd exposure with an increased risk for cancers of the breast, lung, prostate, nasopharynx, pancreas, and kidney—with the kidney and liver being especially susceptible organs [45].

#### Overview

A total of 16 studies (12 published reports) were primarily related to method development and validation for measuring exposure to Pb, Hg, Cd, and As in DBS samples. Of these, eight published reports were from the US, two were from Canada [46, 47], one was from Canada/Tanzania [48], and one was from Germany [49]. Seven studies used NDBS samples. One study compared NDBS measurements to paired whole blood levels [50], one study compared NDBS measurements with cord blood [51], and six studies compared DBS measurements with gold standard venous blood values [46–48, 52].

#### Methods development

The historical development of DBS assays that quantify Pb and other trace elements has been succinctly summarized by a recent review [18]. Here, we will highlight the main developments that apply to Pb, Hg, Cd, and As before discussing each individually. Because standard filter paper used for collecting DBS samples is not designed for trace elemental analyses, contamination is a concern. Trace element contamination can be inherent in the filter paper matrix, and can also occur before, during, and after the blood is collected on the filter paper [52]. In addition, trace element contamination is not homogenously distributed across the card, and therefore performing blank filter paper subtractions using sections of the filter paper adjacent to the blood spot does not work well with low levels of environmental exposure [52, 53]. To address this issue, Funk et al. pretreated the filter paper using a combination of acids to remove contamination prior to DBS sample collection, which vastly improved the agreement between DBS measurements and matched “gold standard” venous blood samples for Pb, Hg, Cd, and As [52]. While this approach cannot be applied when using existing stored samples (e.g., NDBS), it can be used in prospective studies [52]. Funk et al. also evaluated analyte stability and recovery across collection years and found no significant effects of storage time on recovery rates for Pb, Hg, Cd, and As among archived NDBS samples [53].

#### Lead

Recently developed assays for measuring Pb in DBS samples have improved upon prior methods [54–61]. Nyanza et al. developed and validated the most sensitive methods for measuring Pb in field-collected DBS samples using ICP-MS [48]. This study reported a method detection limit (MDL) of 0.08 μg/dL and had a detection frequency of 100% in a sample of 42 pregnant women exposed to high levels from artisanal and small-scale gold mining (ASGM) activities in Tanzania [48]. As noted by Parsons et al. [18], this study was especially impressive for its direct comparison of venous blood and DBS sample values and for its high level of agreement (*R* = 0.969) [48]. The study included both field and laboratory filter blanks to account for potential contamination, and reported field contamination about twice as high as laboratory contamination (0.02 μg/dL versus 0.009 μg/dL) [48], which is less than previously reported contamination levels (between 0.082 and 0.189 μg/dL) [52, 62]. This method had excellent reliability (intraclass correlation was 0.99 for repeated analyses of samples conducted on different days) [48]. DBS samples were stored at room temperature in a desiccator using trace metal-free Nalgene resealable plastic bags for 1–2 weeks prior to shipment to the laboratory [48]. The assay used full punch sizes of 8-mm diameter [48], which improved analytical sensitivity but limits the ability to perform further analyses using the same DBS samples due to finite sample quantity.

Rodríguez-Saldaña et al. validated an assay for quantifying Pb levels in DBS samples using total reflection X-ray fluorescence (TRXF) [46]. The LOD and LOQ for this assay were determined to be 0.28 and 0.69 µg/dL, respectively [46]. Using whole blood reference materials, this assay was determined to have a mean accuracy of 111.1% (97.0–129.7%) and a precision of 14.9% (<15% predefined acceptance criteria) [46]. Internal blanks were analyzed in 14% of the samples, and background Pb levels were essentially negligible [46]. This finding corroborates the low contamination levels reported by Nyanza et al. [48]; however, Funk et al. reported a median of 0.57 µg/dL [53] and geometric mean of 0.189 µg/dL [52] Pb in filter paper blanks. In the study by Rodríguez-Saldaña et al., there was a high level of agreement between TRXF-measured DBS values and venous blood values measured by ICP-MS as assessed by Bland-Altman analyses when applied to a low-exposure group (41 university students) and a relatively high-exposure group (40 electronic waste workers) [46]. Only 7.5% of the samples from the low-exposure group fell below the detection limit, while no samples were below the detection limit in the high-exposure group [46]. In addition, blank filter papers were analyzed from the high-exposure group, since these were collected from a contaminated field site, and no significant field contamination was found [46].

Specht et al. utilized energy-dispersive X-ray fluorescence (EDXRF) to measure the concentration of Pb from 22 DBS samples [63]. Here, Pb levels showed excellent agreement between EDXRF and atomic absorption spectroscopy (*R* = 0.98) [63]. The major advantages of using this EDXRF approach include [1] essentially avoiding potential effects of hematocrit since it is a measurement of the whole blood spot and [2] since EDXRF is a non-destructive process, DBS samples can be saved for further analyses [63]. The LOD of this method was 1.7 μg/dL blood [63], which is significantly higher than the reported detection limits of Rodríguez-Saldaña et al. [46] and other methods. However, increasing the power of the EDXRF system and employing longer measurement times (e.g., >30 min) may further decrease the detection limits and improve analytical sensitivity and precision in future studies [63].

#### Mercury

DBS assays for measuring mercury continue to evolve and there are several recent assays developed for ICP-MS [48, 52, 53], gas chromatography-cold vapor atomic fluorescence spectrometry (GC-CVAFS) [47, 64], and direct Hg analysis [49].

In addition to pre-treating filter paper cards to remove contamination, as discussed previously, Funk et al. demonstrated that the correlation between Hg in archived NDBS and paired filter blanks was significant (*R* = 0.44), suggesting that pair-wise blank subtractions may improve NDBS estimates [53]. However, in a subsequent study, which compared matched venous blood in trace metal-free vacutainers to prospectively collected DBS samples, it was determined that the use of filter paper blanks for background subtraction at the individual level does not work well for quantifying low levels of environmental exposure [52]. In addition, Funk et al. added gold to amalgamate Hg during blood extraction to provide higher extraction efficiency and prevent Hg loss throughout the analytical process [52]. Hg carryover between samples can also be avoided by introducing a wash step consisting of 5% HNO_3_ and 0.5% HCl solution between each ICP-MS run [52]. Nelson et al. also developed an ICP-MS assay for measuring total Hg (T-Hg) in DBS samples and reported an MDL for T-Hg of 0.7 µg/L, compared to 0.3 µg/L for cord blood, in a small cohort (*n* = 48) of urban Minnesota mothers and infants [51]. Because of this higher MDL, Hg exposure was detected in only 38% of NDBS samples compared to 62% of matched cord blood samples [51]. While T-Hg measurements in NDBS samples were highly correlated with matched cord blood samples, NDBS measurements were approximately 15% lower, on average [51].

The DBS assay developed by Nyanza et al. measured T-Hg using ICP-MS and had an MDL for T-Hg was 0.012 µg/L [48]. While using blank filter paper subtraction does not work well with low environmental exposures levels [52], performing blank subtractions in DBS samples collected from a high Hg exposure group may yield better results (although Hg contamination of field and laboratory blanks were quite low in this study, with a mean of 0.006 and 0.003 µg/L, respectively) [48]. After performing blank subtractions, Hg measurements in DBS were highly correlated with venous blood values and were found to be highly reliable with high intraclass correlations and repeatability between duplicate samples [48]. The correlation between DBS and venous blood measurements for Hg was higher than in previously reported studies [52], which the authors suggest could be due to previous studies mixing nitric acid (HNO_3_) and hydrochloric acid (HCl) during the digestion process [48].

Basu et al. developed and validated a highly-sensitive method using GC-CVAFS to quantify methyl mercury (Me-Hg) in NDBS samples [64]. This method had significantly improved analytical sensitivity and precision (LOQ of 0.3 µg/L) and was applied in a relatively large sample of NDBS samples from the Michigan BioTrust cohort (*n* = 675) [64]. Although NDBS measurements were not matched with cord blood data, the DBS values were within the expected range compared to other studies [64]. Hematocrit was investigated by Nelson et al. [51], in which no effects were found, but these analyses were limited by a small sample size [51].

Santa-Rios et al. [47] expanded on this DBS assay [64] to measure Me-Hg and inorganic Hg (I-Hg) in DBS samples using GC-CVAFS. This assay had an excellent agreement between DBS (capillary blood) and paired venous whole blood measures (*R*^2^ = 0.80), reported an MDL of 0.3 µg/L, and used a controlled sample volume (40 µL) in whole DBS spots to minimize potential hematocrit effects [47]. Moreover, Me-Hg measurements in DBS were found to be relatively stable for a 1-year storage period under room temperature conditions [47]. Of note, the previous assay developed by Basu et al. [64] used smaller sample volume requirements (estimated 3.1 µL) and achieved similar sensitivity and precision for detecting Me-Hg. Overall, these studies validated the use of DBS for Me-Hg quantification [47, 64], but quantification of I-Hg using this method had inadequate assay detection limits and requires further development [47]. It is worth noting that when analyzing Me-Hg using ICP-MS, chromatographic separation is required, which adds potential complexity to DBS analyses.

An assay has also recently been developed and validated for quantifying T-Hg by Direct Hg analysis based on atomic absorption spectroscopy and used three discs of 0.5 inches (~60 µL blood) [49]. This study demonstrated analyte stability in pre-cleaned glass tubes at 4 weeks and at elevated temperatures (40 °C) [49]. There was a high agreement between venous blood and DBS sample values, and the reported LOD and LOQ were 0.14 and 0.28 μg/L, respectively [49]. In addition, this study investigated the effects of different storage conditions on Hg stability in DBS samples, demonstrating that pre-cleaned glass tubes may be preferred over plastic bags for storing DBS samples for Hg analyses and that samples are stable for at least 4 weeks at both room temperature and at 40 °C [49].

#### Cadmium

DBS assays for measuring Cd have been limited by inadequate assay detection limits and varying degrees of background contamination of filter cards. Chaudhuri et al. used 6.35-mm punches (~11.5 µL blood) to quantify Cd in archived NDBS samples [62]. However, this study demonstrated high background contamination of filter paper cards, which made it difficult to produce reliable results. For example, DBS samples spiked with 0.62 μg/L of cadmium yielded a 53% recovery after performing blank subtractions [62]. Recovery rates were improved (87%) at higher DBS concentrations [62]. The authors concluded that more research was needed into methods development for this element, and additional experimentation investigating stability across time and storage conditions was not performed [62]. Langer et al. reported median background Cd contamination between 0.02 and 0.14 ng/spot across different lots [65]. This study was able to detect Cd in 100% of DBS samples (*n* = 150) at a median concentration of 0.24 ng/spot [65]. However, using different statistical correction methods in a smaller subset of samples (*n* = 15) resulted in Cd being detected in 0% of DBS samples [65]. This finding was somewhat unexplainable, although higher median Cd concentrations were found in adjacent filter blanks for samples detected only by the first statistical correction method used [65].

Funk et al. measured Cd concentrations of 0.2 ppb (0.2 μg/L) in NDBS samples after performing paired filter paper blank subtractions [53]. Cd was detectable in 67% of samples [53]. The correlation between Cd found in filter paper blanks and NDBS samples was significant (*R* = 0.60), suggesting that paired blank subtractions may improve estimates [53]. When filter paper cards were pretreated to remove contamination, NDBS and venous blood Cd values were highly agreeable (*R*^2^ = 0.94) [52]. Nyanza et al. developed a DBS assay for measuring Cd and, importantly, applied it to a high-exposure group [48]. This study found relatively insignificant levels of Cd in field filter blanks (mean = 0.0011 µg/L) and laboratory filter blanks (mean = 0.001 µg/L) [48]. The MDL was determined to be 0.004 µg/L and all DBS samples (*n* = 44) were above the detection limit [48]. The geometric mean DBS value was 0.361 µg/L (compared to 0.387 µg/L venous blood), indicating both high agreement with gold standard and a relatively high level of exposure among the study sample [48].

#### Arsenic

Blood is not a commonly used matrix for measuring As exposure due to its short residence time in the body [66]. Urine is a more commonly used sampling medium to measure As exposure [67, 68]. The assays developed by Funk et al. were the only methods developed to quantify As in DBS samples [52, 53]. The levels of filter paper blank contamination with As were low for most samples; however, spikes in values were observed in a minority of samples, suggesting possible heterogenous contamination of filter paper [52, 53]. In this study, 82% of the NDBS samples (*n* = 49) analyzed were below the detection limit [53]. Concentrations of As were undetectable in all filter paper blanks [53]. Therefore, pair-wise subtractions of filter paper blanks were not deemed necessary for studies interested in only As exposures [53]. Future work should increase the analytical sensitivity and precision of As quantification to reduce the number of non-detectable DBS samples [53].

#### Guidance

Pre-treating filter paper cards to remove trace element contamination prior to blood collection may improve assay performance [52]. Although inherent contamination in filter paper may be low [48], it is not consistent across lots of filter paper and contamination occurring before, during, and after blood collection may be much higher. Accounting for contamination by performing field and laboratory blank subtractions may be reasonable for relatively high-exposure groups [48]. Contamination of filter paper cards during manufacturing, collection, processing, and storing may be problematic for Pb and Cd, and possibly for As, but is less of a concern for Hg [51–53, 62, 65]. Future work should verify the low levels of contamination in filter paper blanks for Pb, Cd, and As reported by Nyanza et al. [48] and Rodríguez-Saldaña et al. [46]. Hg contamination may be introduced at higher storage temperatures depending on the storage container used [49]. As mentioned by Basu et al. [64], future work should address variations in blood spot volumes, perhaps by normalizing other blood constituents, such as potassium levels. Punching near the edge of blood spots may also minimize variation in blood spreading across the card [64].

According to US NHANES (2011–2018) biomonitoring data, the 50th percentile for blood lead levels is 0.46–0.64 μg/dL and the 90th percentile is 0.93–1.34 μg/dL among children ages 6–11 [37]. Therefore, the assays developed by Nyanza et al. (ICP-MS) [48] and Rodríguez-Saldaña et al. (TXRF) [46] have adequate analytical sensitivity and precision to detect and quantify these levels of lead exposure, with detection limits of 0.08 and 0.28 μg/dL, respectively. In contrast, the DBS methods developed by Specht et al. (EDXRF) [63] (detection limit of 1.7 μg/dL) will need further development to adequately characterize lead exposures in the general population. However, this assay has the major benefit of being non-destructive.

For biomonitoring of T-Hg and Me-Hg in the general population, current DBS assays similarly appear to have sufficient detection limits to characterize exposures. For example, using US NHANES (2011–2018) biomonitoring data, Me-Hg concentrations were 0.39–0.48 µg/L (50th percentile) and 2.23–2.81 µg/L (90th percentile) [37]. Therefore, Basu et al. [64] and Santa-Rios et al. [47] (using GC-CVAFS) report sufficient Me-Hg detection limits of ~0.3 µg/L. Similarly, blood T-Hg concentrations in the population were 0.58–0.64 µg/L (50th percentile) and 2.52–2.87 µg/L (90th percentile) compared to detection limits of 0.012 µg/L reported by Nyanza et al. [48] (ICP-MS) and 0.14 µg/L reported by Schweizer et al. [49] (Direct Hg analysis).

Although the MDL (0.004 µg/L) reported by Nyanza et al. [48] is sufficient to characterize exposures to Cd in the general US population (50th percentile: 0.22–0.25 µg/L, 90th percentile: 0.81–0.96 µg/L) [37], more research is needed to verify these detection limits given varying levels of accuracy, precision, and sensitivity in prior DBS assays [52, 53, 62, 65]. Similarly, more work is needed to sufficiently quantify As in populations with no known exposures.

#### Applications

In a cross-sectional study with a total of 1056 participants (part of the ongoing Mining and Health prospective longitudinal study), Nyanza et al. used DBS sampling to demonstrate that blood T-Hg levels in pregnant women were elevated in those who lived in ASGM communities, compared to a non-ASGM cohort, in Northern Tanzania (50th percentiles: 1.2 versus 0.66 µg/L and 75th percentiles: 1.86 versus 1.2 µg/L) [14]. Spot urine samples were used instead of DBS to estimate As exposure [14]. These findings were later extended to show that elevated blood T-Hg in DBS samples among pregnant women in ASGM communities were significantly associated with stillbirths and visible congenital anomalies [15]. In this same cohort, Nyanza et al. analyzed the associations between T-Hg, T-Pb, and T-Cd measured in maternal DBS samples (collected during weeks 16–27 of pregnancy) and neurodevelopmental outcomes in infants at 6 and 12 months of age [16]. These analyses included 439 mother–infant pairs, since they excluded maternal–infant pairs previously determined to have adverse birth outcomes [15] or lost to follow-up. The results demonstrated that high prenatal exposure to T-Hg was associated with neurodevelopmental and language impairments [16]. While prenatal exposures to high levels of Pb or As were not by themselves associated with neurodevelopmental impairments, prenatal co-exposure to high levels of T-Hg with elevated levels of Pb or As was associated with impairments in neurodevelopment, suggesting synergistic or additive effects [16].

Santa-Rios et al. extended their assay to measure both I-Hg and Me-Hg in DBS samples collected from ASGM and nearby Columbian communities using a cross-sectional study design (*n* = 35) [69]. T-Hg was measured from urine samples, which has been previously validated in exposure assessments [69]. The study used both field and laboratory blanks to account for potential contamination. In this study, only one and four samples were below the previously reported [47] detection limits for Me-Hg and I-Hg, respectively [69]. Field blanks had estimated contamination levels of ~0.07 and ~1.16 µg/L for Me-Hg and I-Hg, respectively [69]. Laboratory blanks had estimated contamination levels of ~0.15 and 1.77 µg/L for Me-Hg and I-Hg, respectively [69]. Me-Hg (%) speciation ranged from 5 to 100%, suggesting that future studies should continue to speciate T-Hg to more clearly identify sources of Hg exposure [69].

Santa-Rios et al. also extended their assay [47] to measure Me-Hg in DBS samples collected from electronic waste workers (*n* = 20) in Ghana [70]. DBS samples and venous blood were collected from the same study participants. DBS samples were also artificially created in the laboratory using collected venous blood samples. T-Hg was measured in venous blood samples. Only one sample fell below the MDL for Me-Hg [70]. There was excellent agreement between Me-Hg values measured in field-collected DBS samples, artificially created DBS samples, and gold standard venous blood samples [70]. Average Me-Hg concentrations were ~0.84 µg/L and Me-Hg speciation was 61% [70]. Me-Hg contamination of field blanks was low [70], corroborating prior studies.

Overall, quality and performance parameters for both application studies conducted by Santa-Rios et al. [69, 70] confirmed that their previously developed DBS methods [47] for measuring Me-Hg meet high-quality standards and are ready for deployment in larger-scale field- and population-based studies, including in contaminated field settings. However, future field-based studies should continue to report background contamination levels by using laboratory and field blanks.

In two studies by Sen et al. DBS sampling was applied to measure early-life exposure to Pb and associated epigenetic alterations [71, 72]. These studies used 3-mm punches and ICP-MS analyses for measuring blood Pb levels in DBS samples [71, 72]. DNA was isolated from the same DBS samples to characterize epigenetic profiles [72]. This group also analyzed associations between a mother’s archived NDBS and the child’s NDBS (collected from the Michigan Neonatal Biobank) to demonstrate that maternal Pb exposure during pregnancy can result in epigenetic alterations in grandchildren (i.e., multigenerational) [71]. Another study similarly used archived NDBS samples from Michigan to demonstrate that elevated newborn exposure to Pb was associated with greater epigenetic alterations, most prominently in pathways related to neurodevelopment [73]. This study used 3-mm DBS punches and reported an MDL of 0.7 µg/L [73]. Out of 129 samples, 21 were below the MDL [73]. The researchers highlight the unique utility of archived NDBS and prospectively collected DBS samples on accelerating the science of environmental epigenetics [73].

### Industrial chemicals

#### Endocrine-disrupting chemicals and persistent organic pollutants

EDCs during the early stages of development can disrupt normal developmental patterns and may have low-dose and non-monotonic effects [74]. EDC exposure is associated with altered reproductive function, thyroid disruption, increased incidence of hormone-related cancers, abnormal growth patterns, neurodevelopmental disorders, and weakened immune systems [75–77]. EDCs include synthetic chemicals used as industrial solvents/lubricants, plastics, pesticides, and pharmaceutical agents [78]. Bisphenol A (BPA) can be found in consumer food and beverage products due to leaching from tinned containers [79]. BPA has been extensively studied and has been found in breast milk, amniotic fluid, and placental tissue [79]. BPA, a xenoestrogen, may have a role in reproductive cancers and fertility issues [79]. BPA has been phased out from most consumer containers and has been banned from infant products [80]. However, the safety profile of bisphenol analogs used as a replacement for BPA has not been well characterized [80]. Perfluorooctanesulfonate (PFOS) and perfluorooctanoate (PFOA), considered per- and polyfluoroalkyl substances (PFASs), are two other EDCs that have been extensively studied. PFOS and PFOA have recently become chemicals of interest after being found in drinking water in communities across the US [81].

POPs are chemicals that persist for long periods in the environment and can accumulate vertically in the food chain due to their ability to remain in adipose tissue [82, 83]. POPs include polychlorinated biphenyls (PCBs), PFASs, polybrominated diphenyl ethers (PBDEs), and organochlorine pesticides [84]. Several POPs, such as PCBs and PFASs, are also considered to be EDCs. PCBs have been associated with cancer and immune, reproductive, nervous system, endocrine system, and metabolic dysfunction [83, 85]. Although policy regulation has led to a decrease in exposure to chlorinated POPs among the general population, exposure to brominated POPs remains widespread [83]. Human exposure to POPs occurs primarily via the consumption of fatty animal-based foods [83]. Biomagnification can lead to human exposure several orders of magnitude greater than levels found in the environment, while its storage in adipose tissue leads to chronic endogenous exposure throughout the lifespan as it is continuously released from adipose tissue [83]. The persistent nature of POPs and their associated health effects make measuring and reducing exposure, especially among infants and children, a key public health concern.

#### Overview

A total of eight studies (five published reports) were primarily related to methods development and validation for measuring exposures to EDCs/POPs in DBS samples. Of these, four reports were from the US [86–89] and one was from Norway [90]. Three studies applied these methods to measure analytes in archived NDBS samples [86, 88, 89]. Two studies compared paired venous blood values to DBS measurements [87, 90].

#### Methods development

Barr et al. recently reviewed several DBS assays for measuring EDCs and POPs from a laboratory-based perspective, and suggested future considerations for improving the methods and reliability of DBS sampling for measuring these exposure biomarkers [17]. Here, we highlight the most well-developed and validated assays and their applications to population-based studies.

Ma et al. developed and validated methods for quantifying EDCs in DBS samples utilizing high-performance liquid chromatography (HPLC) and tandem MS to detect PFOS, PFOA, and BPA in 16-mm NDBS samples containing approximately 50 μL of blood [86]. Recovery rates from spiked samples were 79 and 92% for PFOS and PFOA, respectively, while BPA had a recovery rate of 39% [86]. Background levels of PFOS and PFOA were of minimal concern with trace amounts, 0.01 and 0.1 μg/L, respectively, found in filter paper blanks [86]. This contamination was thought to be from the reagents used and not from the filter paper itself. However, background levels of BPA in filter paper may be significant (0.5–0.8 μg/L) and should be taken into consideration [86]. PFOS had the lowest LOD at 0.03 μg/L (LOQ 0.1 μg/L), followed by PFOA at 0.05 μg/L (LOQ 0.2 μg/L), and BPA at 0.3 μg/L (1.0 μg/L) [86].

The method was applied to 192 NDBS samples from infants born in New York between 2008 and 2011 [86]. PFOS and PFOA were detected in 100% of samples analyzed with concentrations ranging from 0.27 to 6.46 μg/L and 0.21 to 4.35 μg/L, respectively [86]. Serum reference ranges among adolescents (ages 12–19) in the US (NHANES 2011–2018) were 2.60–4.11 μg/L (50th percentile) and 11.5–15.7 μg/L (90th percentile) for PFOS and 1.17–1.74 μg/L (50th percentile) and 2.07–2.93 μg/L (90th percentile) for PFOA [37]. BPA was found in 86% of samples at concentrations ranging from 0.2 to 35 ng/mL [86]. Field blanks were used to demonstrate that there was little contamination introduced during collection, storage, and shipping [86].

Poothong et al. developed a reliable method to measure a range of PFASs in human 3-mm punch DBS blood samples (~3.3 μL blood) from 59 Norwegian adults using an online solid phase extraction, ultra-high-performance liquid chromatography with tandem mass spectrometry (online SPE-UHPLC-MS/MS) quantification method [90]. For gold standard comparisons, 10 punches were used (~33 μL blood) and compared to whole DBS spots (~50 μL blood). These analyses demonstrated strong agreement between finger-prick DBS and venous whole blood samples (*R* = 0.72) [90]. The reported MDLs ranged from 0.008 to 0.3 μg/L, which were comparable to Ma et al. [91]. The study also did not find any significant effects of hematocrit on PFAS measurements [90]. Of the 25 PFASs measured in paired DBS and whole blood samples, only seven (perfluorohexane sulfonate (PFhxS), PFOS, PFOA, perfluorononanoic acid (PFNA), PFDA, PFUnDA, and perfluorooctane sulfonamide (PFOSA)) had satisfactory detection frequencies (>85%) and were used in further statistical analyses [90].

Batterman and Chernyak used GC-MS to measure 11 compounds including PCBs, PBDEs, and persistent pesticides in adult DBS samples [87]. The study found strong agreement between 50 μL DBS and whole blood samples from six volunteers [87]. Furthermore, sample integrity remained high in storage extending up to 1 year when samples were stored at refrigerated or frozen temperatures [87]. However, when stored at room temperature, sample integrity was high for up to 1 month [87]. Kato et al. also demonstrated the stability of several POPs in NDBS samples when stored at 37 °C for 61 days [88]. Batterman and Chernyak reported consistent background contamination of several POPs in DBS samples [87]. This contamination was confirmed to originate from the blank filter paper and not from the extraction or sample processing methods [87]. No additional contamination was observed as a function of storage time [87].

#### Applications

Spliethoff et al. used HPLC for temporal biomonitoring of PFOS, PFOSA, PFHxS, PFOA, and PFNA in 110 pooled composite DBS samples representing 2640 infants from New York State between 1997 and 2007 [92]. All analytes were detected in ≥90% of specimens and concentrations of PFOS, PFOSA, PFHxS, and PFOA decreased significantly after the year 2000, coinciding with the phasing out of PFOS production in the United States [92]. These methods were validated using spiked venous blood samples from adult volunteers [92]. Recoveries ranged from 60 to 112%, suggesting a slight bias toward lower values overall [92]. Field blanks were used to measure and adjust for background contamination present in the filter paper [92]. This study demonstrated the validity and efficacy of using pooled DBS sampling for temporal biomonitoring.

In two separate studies, Ma et al. used gas chromatography-high-resolution mass spectrometry for temporal biomonitoring by measuring exposure to POPs in 51 blood spot composites from 1224 newborns [91, 93]. The mean whole blood concentration of PCBs in Upstate New York newborn blood samples was found to be 1.06 ng/mL between 1997 and 2011, with a significant decrease between 1997 and 2001 and no significant reduction thereafter [91]. Ma et al. also observed mean concentrations of 0.128 ng/mL for PBDE congener brominated diphenyl ethers (BDE)-47, 0.040 ng/mL for BDE-99, and 0.012 ng/mL for BDE-100 [93]. Both studies used pooled blood spot composites resulting in a total estimated blood volume of 322 μL per sample [93]. The methodology was validated using spiked DBS samples at 0.2 and 2 ng/mL for each target compound [91, 93]. The PBDE congener recoveries ranged between 53.7 and 79.0% at the 0.2 ng/mL concentration and from 73.0 to 85.7% at the 2 ng/mL concentration. Consequently, PCB recoveries ranged between 51.8 and 102% at the 0.2 ng/mL concentration and from 89.2 to 114% at the 2 ng/mL concentration.

Several studies have applied the validated assay [86] developed by Ma et al. to measure concentrations of PFOS, PFOA, and BPA in archived NDBS samples collected from the Upstate KIDS Study (New York). Bell at el. measured PFOS, PFOA, and BPA in 3111 samples from singleton and twin infants and their relationship with infant health outcomes [94]. The study found that PFOS and PFOA levels were above detectable limits in >99% of samples and in 90% of samples for BPA [94]. The study observed no significant associations between PFAS and birth size controlling for plurality of birth, while BPA was negatively associated with birth size in twins [94]. In another analysis of the same NDBS data (*n* = 3111), Yeung et al. analyzed the association between newborn exposure to these EDCs and early childhood growth patterns, including weight gain and obesity rates. PFOS and PFOA values were highly correlated (*R* > 0.75) in NDBS samples from related twins; however, the association was lower for BPA (*R* = 0.23) [95]. The study suggested that newborn exposure to BPA may occur through extended hospital stays in the neonatal intensive care unit [95]. BPA measured in NDBS samples may therefore represent postnatal exposures (e.g., from medical devices) as opposed to prenatal exposures [95].

In the same study population and NDBS data, Ghassabian et al. assessed the relationship between PFOS, PFOA, and BPA and children’s behavior at 7 years [96]. In this analysis, 100% of specimens had detectable levels of PFOS and PFOA while BPA was detected in 86% of the specimens [96]. The differences in detection frequencies can be attributed to the smaller sample size used (*n* = 788 or 918 depending on the analysis). The study concluded that higher PFOS levels were associated with increased odds of behavioral difficulties, while increased PFOA was associated with difficulties in prosocial behaviors [96]. Neonatal BPA levels measured in NDBS, on the other hand, were not clearly associated with increased behavioral difficulties [96]. Another analysis of data from the Upstate KIDS Study found higher concentrations of some POPs associated with a small increased risk for gestational age and birth weight [97]. This study also demonstrated the potential utility of pooling DBS samples for increasing assay detection limits [97]. Most recently, Robinson et al. analyzed NDBS data (*n* = 597) from the Upstate KIDS Study for associations between PFOS and PFOA levels and epigentic alterations [98]. DNA was extracted from the NDBS samples using three discs of 0.5 inches [98]. Gross et al. also recently used NDBS samples to investigate the association between neonatal exposures to POPs and overweight status in a nested case–control study including a low-income Hispanic urban population [99]. Overall, these studies support the feasibility and utility of EDC quantification using residual NDBS samples.

### Other environmental exposure biomarkers

Due to space limitations, we have not discussed here DBS assays to measure environmental exposures to benzene [100], fipronil (insecticide) [101], parabens [102], and acrylamide [103]. However, these assays are included in Table 1.

## Discussion

In this review and guide for using DBS sampling in population-based research, we provide a summary of DBS assays that have been developed and validated for measuring exposure biomarkers for investigators that are collecting, or planning to collect, DBS samples to investigate environmental causes of disease. The use of DBS sampling to estimate environmental exposures to chemical toxicants provides a simple and non-invasive means for obtaining blood samples in population-based studies, which is particularly well suited for field-based studies conducted in low-resource settings and in large cohort studies involving infants and children. Recent improvements in analytical sensitivities have vastly reduced blood volume requirements allowing for accurate detection and quantification of an array of exposure biomarkers. Together, these advancements provide extensive opportunities for investigating links between environmental exposures and adverse health outcomes.

High-performing DBS methods have been developed, validated, and applied for measuring exposures to ETS (cotinine), trace elements (e.g., Pb and Hg), and several important EDCs and POPs. In addition, DBS assays tend to show high correlations with gold standard venous blood assays for many exposure biomarkers, including cotinine, lead, total mercury, methyl mercury, and several EDCs and POPs. As a result, DBS sampling may be an attractive option in epidemiological studies measuring these biomarkers when venous blood collection is not feasible. In addition, DBS sampling presents a unique opportunity to advance environmental epigenetics, especially among hard-to-reach populations [71–73].

However, uncertainties remain regarding background contamination levels in filter paper, especially for Pb, As, Cd, and BPA. Additional work is also needed to improve the MDLs (i.e., sensitivity and precision) of assays for measuring As, Cd, and BPA before their widespread use in large-scale population-based studies. Future method development studies should ensure consistent evaluation and reporting of key quality-control assay parameters, including precision, reliability, accuracy/recovery, sensitivity, stability, and detection frequencies [9, 17], to accelerate improvements in analytical performance and facilitate comparisons between assays. Studies applying previously developed DBS methods to population-based studies should continue to report quality assurance parameters and should perform method and field blank subtractions to facilitate DBS sampling as a reliable tool for advancing public health and environmental epidemiology. In addition, the DBS assays discussed here have not yet been reliably reproduced across different laboratories, which would be a major next step in validation [54].

The implementation of DBS sampling in low- and middle-income countries may be enhanced by existing public health infrastructure that collects DBS samples for other purposes, such as for the monitoring of antiretroviral treatment among HIV-positive patients (i.e., viral load measurements), surveillance of HIV drug resistance, expansion of early infant diagnosis of HIV programs, or for malaria diagnostic testing [48, 69, 70, 104–109]. Another promising avenue of future research is the prospect of enabling study participants to self-collect DBS samples [10, 11]. With the persistence of the COVID-19 pandemic, widespread collection of DBS samples are being incorporated into community- and hospital-based seroprevalence studies, which use DBS sampling to detect the levels of SARS-CoV-2 IgG antibodies [110–112]. Future research may use residual DBS samples collected for the purposes of seroprevalence studies for measuring exposure biomarkers among subpopulations of interest.

In addition, because of the COVID-19 pandemic, many existing environmental health cohort studies have been disrupted and have not been able to collect blood samples from study participants as planned (e.g., ECHO cohorts). As an alternative method for measuring exposure biomarkers with well-developed and validated assays, self-collection of DBS samples may be a feasible method for continuing to obtain blood samples during potentially critical developmental periods for study participants. However, contamination remains a significant issue for many target analytes in DBS samples. Therefore, contamination may be a concern with the self-collection of DBS samples by untrained study participants, which will need to be addressed in future investigations.

## Supplementary information

Supplementary Information

Reporting Checklist
