# Correction to: A state-of-the-science review and guide for measuring environmental exposure biomarkers in dried blood spots

**DOI:** 10.1038/s41370-022-00488-9

**Published:** 2022-11-02

**Authors:** Tyler A. Jacobson, Jasdeep S. Kler, Yeunook Bae, Jiexi Chen, Daniel T. Ladror, Ramsunder Iyer, Denise A. Nunes, Nathan D. Montgomery, Joachim D. Pleil, William E. Funk

**Affiliations:** 1 Department of Preventive Medicine, Northwestern University Feinberg School of Medicine, Chicago, IL USA; 2 University of Michigan Medical School, Ann Arbor, MI USA; 3 Galter Health Sciences Library, Northwestern University Feinberg School of Medicine, Chicago, IL USA; 4 Department of Environmental Sciences and Engineering, Gillings School of Public Health, University of North Carolina, Chapel Hill, NC USA

Correction to: *Journal of Exposure Science and Environmental Epidemiology* 10.1038/s41370-022-00460-7

The article “A state-of-the-science review and guide for measuring environmental exposure biomarkers in dried blood spots”, written by Tyler A. Jacobson, Jasdeep S. Kler, Yeunook Bae, Jiexi Chen, Daniel T. Ladror, Ramsunder Iyer, Denise A. Nunes, Nathan D. Montgomery, Joachim D. Pleil and William E. Funk, was originally published electronically on the publisher’s internet portal on 13 August 2022 without open access. With the author(s)’ decision to opt for Open Choice the copyright of the article changed on 18 October 2022 to © The Author(s) 2022 and the article is forthwith distributed under a Creative Commons Attribution 4.0 International License, which permits use, sharing, adaptation, distribution and reproduction in any medium or format, as long as you give appropriate credit to the original author(s) and the source, provide a link to the Creative Commons licence, and indicate if changes were made. The images or other third party material in this article are included in the article’s Creative Commons licence, unless indicated otherwise in a credit line to the material. If material is not included in the article’s Creative Commons licence and your intended use is not permitted by statutory regulation or exceeds the permitted use, you will need to obtain permission directly from the copyright holder. To view a copy of this licence, visit http://creativecommons.org/licenses/by/4.0/.

The original article has been corrected.

