# Supplementary material for: A state-of-the-science review and guide for measuring environmental exposure biomarkers in dried blood spots
Source: J Expo Sci Environ Epidemiol. 2022 Aug 13;33(4):505–23. doi: 10.1038/s41370-022-00460-7 (PMC9375076; doi:10.1038/s41370-022-00460-7)
Supplement: Supplementary file 1 — Supplementary Information [file 41370_2022_460_MOESM1_ESM.docx]

**Supplementary Information: A State-of-the-Science Review and Guide for Measuring Environmental Exposure Biomarkers in Dried Blood Spots**

## **S1. METHODS:**

***Inclusion/Exclusion Criteria:***

To be included in this review, studies had to meet the following criteria:

- Measured environmental exposure biomarker (i.e., internal doses of exposure to exogenous pollutants, including native exogenous compounds and/or their metabolites).
- Used dried blood spot (DBS) sampling.
- Used human blood (not animal blood).

DBS assays developed for measuring exposure to infectious diseases, drugs, alcohol, and endogenously produced chemicals were excluded. Studies which only measured biological response markers were excluded, including erythrocyte arginase activity and cholinesterase depression, since these are primarily used in hazard assessment as a mechanism of toxicity. We also excluded methods for measuring exposure to chemical warfare agents since these are not typically considered environmental exposures. Five published reports related to measuring exposure biomarkers to mycotoxins in human DBS samples were excluded in this review due to space limitations and because these exposures are more biological in nature. These reports will be summarized in a separate forthcoming review.

***Additional search strategy methods and limitations:***

We searched the databases listed below on **March 7, 2022.** Records from each database were exported to EndNote. Duplicates were removed through the “Find Duplicates” function within EndNote and were manually reviewed. Search strategies from each of the bibliographic databases are available below. Although our systematic search did not apply a date filter, we excluded studies published prior to 1990 since these assays were deemed to be outdated and the purpose of the present review was to focus on more recently developed DBS methods.

Our search strategy has the following limitations. First, we searched terms as subject headings or within the title/abstracts. Therefore, if an article discussed DBS and environmental exposure biomarkers (or related search terms) but did not include these terms in the Title or Abstract, or if the article was not indexed with compatible subject headings in the database (e.g., MESH terms), it may not have been captured. In general, older articles are not indexed as extensively in databases and are therefore more likely to be missed by our systematic search. In addition, specific exposure biomarkers were not included as search terms since this would bias our search results and we were interested in mapping which environmental exposure biomarkers had existing assays adapted for DBS analyses.

## **March 2022 Database Systematic Search Overview**

| Database | Coverage | Date Searched | Results |
| --- | --- | --- | --- |
| PubMed (NIH/NLM)   - MEDLINE (1946 to present) - PubMed Central (1700 to present) - Bookshelf (N/A) | 1700 to present | 3/07/2022 | 973 |
| Embase (Elsevier) | 1947 to present | 03/07/2022 | 1493 |
| CINAHL Plus with Full Text (EBSCOhost) | 1937 to present | 3/07/2022 | 149 |
| Total | |  | 2615 |
| Total After De-Duplication | |  | 1620 |

# PubMed

| Line | Query | Results |
| --- | --- | --- |
| 1 | Dried Blood Spot Testing[Mesh] | 1,917 |
| 2 | dbs[Title] | 920 |
| 3 | ("Guthrie card*"[Title/Abstract] OR "Guthrie paper*"[Title/Abstract] OR "dried blood"[Title/Abstract] OR "dried whole blood spot*"[Title/Abstract]) | 6,337 |
| 4 | (("blood blot*"[Title/Abstract] OR "blood spot*"[Title/Abstract] OR bloodspot*[Title/Abstract]) AND "filter paper*"[Title/Abstract]) | 1,022 |
| 5 | #1 OR #2 OR #3 OR #4 | 7,797 |
| 6 | "Biomarkers"[Majr] | 284,140 |
| 7 | Environmental Biomarkers [Mesh] | 585 |
| 8 | (Biomarker*[Title/Abstract] OR "environmental biomarkers"[Title/Abstract] OR "surrogate endpoints"[Title/Abstract]) | 347,466 |
| 9 | (marker*[Title/Abstract] AND (biologic*[Title/Abstract] OR laboratory[Title/Abstract] OR serum[Title/Abstract] OR immun*[Title/Abstract] OR clinical[Title/Abstract] OR biochemical[Title/Abstract] OR viral[Title/Abstract])) | 455,613 |
| 10 | #6 OR #7 OR #8 OR #9 | 935,945 |
| 11 | Environmental Pollutants[Majr] | 236,295 |
| 12 | Environmental Exposure[Mesh] | 331,330 |
| 13 | Carcinogens, Environmental[Mesh] | 4,511 |
| 14 | Exposome[Title/Abstract] OR "biological monitoring"[Title/Abstract] OR "bio-monitoring"[Title/Abstract] | 4,203 |
| 15 | Environment*[Title/Abstract] AND ( exposur*[Title/Abstract] OR pollutant*[Title/Abstract] OR monitor*[Title/Abstract] OR surveillance[Title/Abstract] OR carcinogen[Title/Abstract]) | 236,984 |
| 16 | #11 OR #12 OR #13 OR #14 OR #15 | 636,687 |
| 17 | #5 AND #10 | 863 |
| 18 | #5 AND #16 | 155 |
| 19 | #17 OR #18 | 973 |

# Embase

| No. | Query | Results |
| --- | --- | --- |
| 1 | 'dried blood spot testing'/exp | 5032 |
| 2 | dbs:ti | 2739 |
| 3 | 'guthrie card*':ab,ti OR 'guthrie paper*':ab,ti OR 'dried blood':ab,ti OR 'dried whole blood spot*':ab,ti OR 'dried whole bloodspot*':ab,ti | 9338 |
| 4 | ('blood blot*':ab,ti OR 'blood spot*':ab,ti OR bloodspot*:ab,ti) AND 'filter paper*':ti,ab | 1427 |
| 5 | #1 OR #2 OR #3 OR #4 | 13602 |
| 6 | 'biological marker'/exp/mj | 102408 |
| 7 | 'environmental marker'/exp | 769 |
| 8 | biomarker*:ab,ti OR 'environmental biomarkers':ab,ti OR 'surrogate endpoints':ab,ti | 501499 |
| 9 | marker*:ab,ti AND (biologic*:ab,ti OR laboratory:ab,ti OR serum:ab,ti OR immun*:ab,ti OR clinical:ab,ti OR biochemical:ab,ti OR viral:ab,ti) | 700424 |
| 10 | #6 OR #7 OR #8 OR #9 | 1131802 |
| 11 | 'pollutant'/exp | 366042 |
| 12 | 'environmental exposure'/exp | 118128 |
| 13 | 'carcinogen'/exp | 167798 |
| 14 | exposome:ab,ti OR 'biological monitoring':ab,ti OR 'bio-monitoring':ab,ti | 4907 |
| 15 | environment*:ab,ti AND (exposur*:ab,ti OR pollutant*:ab,ti OR monitor*:ab,ti OR surveillance:ab,ti OR carcinogen:ab,ti) | 286565 |
| 16 | #11 OR #12 OR #13 OR #14 OR #15 | 834443 |
| 17 | #5 AND #10 | 1326 |
| 18 | #5 AND #16 | 218 |
| 19 | #17 OR #18 | 1493 |

# CINAHL

| **#** | **Query** | **Results** |  |
| --- | --- | --- | --- |
| 1 | TI dbs | 312 |  |
|  |  |  |  |
|  |  |  |  |
| 2 | TI ( ("Guthrie card*" or "Guthrie paper*" or "dried blood" or "dried whole blood spot*" or "dried whole bloodspot*") ) OR AB ( ("Guthrie card*" or "Guthrie paper*" or "dried blood" or "dried whole blood spot*" or "dried whole bloodspot*") ) | 978 |  |
|  |  |  |  |
|  |  |  |  |
| 3 | TI ( (("blood blot*" or "blood spot*" or bloodspot*) and "filter paper*") ) OR AB ( (("blood blot*" or "blood spot*" or bloodspot*) and "filter paper*") ) | 83 |  |
|  |  |  |  |
|  |  |  |  |
| 4 | (MM "Biological Markers") | 18,636 |  |
|  |  |  |  |
|  |  |  |  |
| 5 | TI ( Biomarker* OR “environmental biomarkers” OR “surrogate endpoints” ) OR AB ( Biomarker* OR “environmental biomarkers” OR “surrogate endpoints” ) | 66,448 |  |
|  |  |  |  |
|  |  |  |  |
| 6 | TI ( (marker* AND (biologic* OR laboratory OR serum OR immun* OR clinical OR biochemical OR viral)) ) OR AB ( (marker* AND (biologic* OR laboratory OR serum OR immun* OR clinical OR biochemical OR viral)) ) | 59,909 |  |
|  |  |  |  |
|  |  |  |  |
| 7 | (MM "Environmental Pollutants") | 2,777 |  |
|  |  |  |  |
|  |  |  |  |
| 8 | (MH "Environmental Exposure+") | 49,303 |  |
|  |  |  |  |
|  |  |  |  |
| 9 | (MH "Carcinogens, Environmental") | 388 |  |
|  |  |  |  |
|  |  |  |  |
| 10 | TI ( Exposome or “biological monitoring” OR “bio-monitoring” ) OR AB ( Exposome or “biological monitoring” OR “bio-monitoring” ) | 394 |  |
|  |  |  |  |
|  |  |  |  |
| 11 | TI ( Environment* AND ( exposur* OR pollutant* OR monitor* OR surveillance OR carcinogen) ) OR AB ( Environment* AND ( exposur* OR pollutant* OR monitor* OR surveillance OR carcinogen) ) | 27,090 |  |
|  |  |  |  |
|  |  |  |  |
| 12 | S1 OR S2 OR S3 | 1,310 |  |
|  |  |  |  |
|  |  |  |  |
| 13 | S4 OR S5 OR S6 | 124,729 |  |
|  |  |  |  |
|  |  |  |  |
| 14 | S7 OR S8 OR S9 OR S10 OR S11 | 70,593 |  |
|  |  |  |  |
|  |  |  |  |
| 15 | S12 AND S13 | 131 |  |
|  |  |  |  |
|  |  |  |  |
| 16 | S12 AND S14 | 30 |  |
|  |  |  |  |
|  |  |  |  |
| 17 | S15 OR S16 | 149 |  |
|  |  |  |  |
|  |  |  |  |
|  |  |  |  |

## **S2. Blood Volume Estimation Depending on Disc Size of DBS sample:**

***Blood volume corresponding to a half-inch spot in filter card (V_sp_):***

Based on careful evaluations in our lab, we found exactly **50 µL** of blood volume (*V_sp_*) is required to fill the whole blood spot (**Figure 3**). Assuming 50 µL of blood per spot, we made the following calculations, which were used for estimates of whole blood volume sample requirements used in **Table 1**.

***Surface area of spot (half-inch) in the filter paper:***

- Diameter (*D_sp_*, mm): $D_{sp}=0.5 in= 0.5 in\cdot25.4 \frac{mm}{in} = \boldsymbol{12.7 mm}$
- Surface area (*A_sp_*, mm^2^): $A_{sp}=\frac{\pi}{4} \cdot{D_{sp}}^{2}=\frac{\pi}{4} \cdot{(12.7 mm)}^{2}= \boldsymbol{126.7}\boldsymbol{mm}^{\boldsymbol{2}}$

***Blood volume corresponds to a half-inch spot in filter card:***

- 3.0-mm disk (*D_3.0_*, mm):
  - Surface area (*A_3.0_*, mm^2^): $A_{3.0}=\frac{\pi}{4} \cdot{D_{3.0}}^{2}=\frac{\pi}{4} \cdot{(3.0 mm)}^{2}= \boldsymbol{7.1}\boldsymbol{mm}^{\boldsymbol{2}}$
  - Blood volume (*V_3.0_*, µL): $V_{3.0}=V_{sp} \cdot\frac{7.1 {mm}^{2}}{126.7 {mm}^{2}} = \boldsymbol{2.8 \mu}\mathbf{L}$
- 3.2-mm disk (*D_3.2_*, mm):
  - Surface area (*A_3.2_*, mm^2^): $A_{3.2}=\frac{\pi}{4} \cdot{D_{3.2}}^{2}=\frac{\pi}{4} \cdot{(3.2 mm)}^{2}= \boldsymbol{8.0}\boldsymbol{mm}^{\boldsymbol{2}}$
  - Blood volume (*V_3.2_*, µL): $V_{3.2}=V_{sp} \cdot\frac{8.0 {mm}^{2}}{126.7 {mm}^{2}} = \boldsymbol{3.2 \mu}\mathbf{L}$
- 4.7-mm disk (*D_4.7_*, mm):
  - Surface area (*A_4.7_*, mm^2^): $A_{4.7}=\frac{\pi}{4} \cdot{D_{4.7}}^{2}=\frac{\pi}{4} \cdot{(4.7 mm)}^{2}= \boldsymbol{17.3}\boldsymbol{mm}^{\boldsymbol{2}}$
  - Blood volume (*V_4.7_*, µL): $V_{4.7}=V_{sp} \cdot\frac{17.3 {mm}^{2}}{126.7 {mm}^{2}} = \boldsymbol{6.9 \mu}\mathbf{L}$
- 4.76-mm (3/16 inch) disk (*D_4.76_*, mm):
  - Surface area (*A_4.76_*, mm^2^): $A_{4.76}=\frac{\pi}{4} \cdot{D_{4.76}}^{2}=\frac{\pi}{4} \cdot{(4.76 mm)}^{2}= \boldsymbol{17.8}\boldsymbol{mm}^{\boldsymbol{2}}$
  - Blood volume (*V_4.76_*, µL): $V_{4.76}=V_{sp} \cdot\frac{17.8 {mm}^{2}}{126.7 {mm}^{2}} = \boldsymbol{7.0 \mu}\mathbf{L}$
- 4.8-mm disk (*D_4.8_*, mm):
  - Surface area (*A_4.8_*, mm^2^): $A_{4.8}=\frac{\pi}{4} \cdot{D_{4.8}}^{2}=\frac{\pi}{4} \cdot{(4.8 mm)}^{2}= \boldsymbol{18.1}\boldsymbol{mm}^{\boldsymbol{2}}$
  - Blood volume (*V_4.8_*, µL): $V_{4.8}=V_{sp} \cdot\frac{18.1 {mm}^{2}}{126.7 {mm}^{2}} = \boldsymbol{7.1 \mu}\mathbf{L}$
- 6.0-mm disk (*D_6.0_*, mm):
  - Surface area (*A_6.0_*, mm^2^): $A_{6.0}=\frac{\pi}{4} \cdot{D_{6.0}}^{2}=\frac{\pi}{4} \cdot{(6.0 mm)}^{2}= \boldsymbol{28.3}\boldsymbol{mm}^{\boldsymbol{2}}$
  - Blood volume (*V_6.0_*, µL): $V_{6.0}=V_{sp} \cdot\frac{28.3 {mm}^{2}}{126.7 {mm}^{2}} = \boldsymbol{11.2 \mu}\mathbf{L}$
- 6.35-mm (1/4 inch) disk (*D_6.35_*, mm):
  - Surface area (*A_6.35_*, mm^2^): $A_{6.35}=\frac{\pi}{4} \cdot{D_{6.35}}^{2}=\frac{\pi}{4} \cdot{(6.35 mm)}^{2}= \boldsymbol{31.7}\boldsymbol{mm}^{\boldsymbol{2}}$
  - Blood volume (*V_6.35_*, µL): $V_{6.35}=V_{sp} \cdot\frac{31.7 {mm}^{2}}{126.7 {mm}^{2}} = \boldsymbol{12.5 \mu}\mathbf{L}$
- 8.0-mm disk (*D_8.0_*, mm):
  - Surface area (*A_8.0_*, mm^2^): $A_{8.0}=\frac{\pi}{4} \cdot{D_{8.0}}^{2}=\frac{\pi}{4} \cdot{(8.0 mm)}^{2}= \boldsymbol{50.3}\boldsymbol{mm}^{\boldsymbol{2}}$
  - Blood volume (*V_8.0_*, µL): $V_{8.0}=V_{sp} \cdot\frac{50.3 {mm}^{2}}{126.7 {mm}^{2}} = \boldsymbol{19.8 \mu}\mathbf{L}$
- 12.7-mm (1/2 inch) disk (*D_12.7_*, mm):
  - Surface area (*A_12.7_*, mm^2^): $A_{12.7}=\frac{\pi}{4} \cdot{D_{12.7}}^{2}=\frac{\pi}{4} \cdot{(12.7 mm)}^{2}= \boldsymbol{126.7}\boldsymbol{mm}^{\boldsymbol{2}}$
  - Blood volume (*V_12.7_*, µL): $V_{12.7}=V_{sp} \cdot\frac{126.7 {mm}^{2}}{126.7 {mm}^{2}} = \boldsymbol{50.0 \mu}\mathbf{L}$
- 15.0-mm disk (*D_15.0_*, mm):
  - Surface area (*A_15.0_*, mm^2^): $A_{15.0}=\frac{\pi}{4} \cdot{D_{15.0}}^{2}=\frac{\pi}{4} \cdot{(15.0 mm)}^{2}= \boldsymbol{176.7}\boldsymbol{mm}^{\boldsymbol{2}}$
  - Blood volume (*V_15.0_*, µL): $V_{15.0}=V_{sp} \cdot\frac{176.7 {mm}^{2}}{126.7 {mm}^{2}} = \boldsymbol{69.8 \mu}\mathbf{L}$
- 16.0-mm disk (*D_16.0_*, mm):
  - Surface area (*A_16.0_*, mm^2^): $A_{16.0}=\frac{\pi}{4} \cdot{D_{16.0}}^{2}=\frac{\pi}{4} \cdot{(16.0 mm)}^{2}= \boldsymbol{201.1}\boldsymbol{mm}^{\boldsymbol{2}}$
  - Blood volume (*V_16.0_*, µL): $V_{16.0}=V_{sp} \cdot\frac{201.1 {mm}^{2}}{126.7 {mm}^{2}} = \boldsymbol{79.4 \mu}\mathbf{L}$
- 2-mm × 6-mm rectangular punch (d, mm × w, mm):
  - Surface area (*A_2×6_*, mm^2^): $A_{2\times6}=d \cdot w=2.0 mm\cdot6.0 mm= \boldsymbol{12.0}\boldsymbol{mm}^{\boldsymbol{2}}$
  - Blood volume (*V_16.0_*, µL): $V_{16.0}=V_{sp} \cdot\frac{12.0 {mm}^{2}}{126.7 {mm}^{2}} = \boldsymbol{4.7 \mu}\mathbf{L}$
